# Genomic Analysis of Infectious Bursal Disease Virus in Nigeria: Identification of Unique Mutations of Yet Unknown Biological Functions in Both Segments A and B

**DOI:** 10.3390/vaccines11040867

**Published:** 2023-04-19

**Authors:** Ijeoma Nwagbo, Adelaide Milani, Annalisa Salviato, Gianpiero Zamperin, Lanre Sulaiman, Nanven Maurice, Clement Meseko, Alice Fusaro, Ismaila Shittu

**Affiliations:** 1 National Veterinary Research Institute, Vom 930010, Nigeria; ijeoma.nwagbo@nvri.gov.ng (I.N.); clement.meseko@nvri.gov.ng (C.M.); 2 Istituto Zooprofilattico Sperimentale delle Venezie, 35020 Legnaro, Padova, Italy; amilani@izsvenezie.it (A.M.);

**Keywords:** genome sequence, infectious bursal disease virus, Nigeria, phylogenetic analysis, poultry, segments A and B

## Abstract

Infectious bursal disease (IBD) is a viral poultry disease known worldwide for impacting the economy and food security. The disease is endemic in Nigeria, with reported outbreaks in vaccinated poultry flocks. To gain insight into the dynamics of infectious bursal disease virus (IBDV) evolution in Nigeria, near-complete genomes of four IBDVs were evaluated. Amino acid sequences in the hypervariable region of the VP2 revealed conserved markers (222A, 242I, 256I, 294I and 299S) associated with very virulent (vv) IBDV, including the serine-rich heptapeptide motif (SWSASGS). Based on the newly proposed classification for segments A and B, the IBDVs clustered in the A3B5 group (where A3 are IBDVs with vvIBDV-like segment A, and where B5 are from non-vvIBDV-like segment B) form a monophyletic subcluster. Unique amino acid mutations with yet-to-be-determined biological functions have been observed in both segments. Amino acid sequences of the Nigerian IBDVs showed that they are reassortant viruses. Circulation of reassortant IBDVs may be responsible for the vaccination failures observed in the Nigerian poultry population. Close monitoring of changes in the IBDV genome is recommended to nip deleterious changes in the bud through the identification and introduction of the most appropriate vaccine candidates and advocacy/extension programs for properly implementing disease control.

## 1. Introduction

Infectious bursal disease (IBD) is a viral poultry disease that is known worldwide not only for its devastating effects on poultry [1,2] but, also, for its impact on other aspects of human existence vis-a-vis economy and food security. In terms of susceptibility, young chicks between 3 and 6 weeks of age are most susceptible to IBD infections, typically characterized by the destruction of B lymphocytes in the lymphoid organ, resulting in death. Hence, surviving chicks become immunosuppressed, making them susceptible to other diseases [2]. However, infections have been reported in older chickens of up to 20 weeks [3,4].

The aetiologic agent, Infectious Bursal Disease Virus (IBDV), is a non-enveloped, double stranded RNA virus assigned to the family *Birnaviridae*, genus *Avibirnavirus* [5]. The IBDV genome is bi-segmented [6], comprising the larger segment A (3.2 kbp) encoding VP2, VP4, and VP3 [7,8], and the smaller overlapping open reading frame encoding a non-structural protein, VP5 [9,10]. The VP2, the capsid protein, is located on the surface of the virus and within the VP2 are sites responsible for virus neutralization, viral virulence, and antigenic variation. The VP4 is the viral protease involved in the cleavage of the polyprotein (NH2-VP2-VP4-VP3-COOH), while the VP3 is involved in the morphogenesis and encapsidation of the viral genome [7,8]. The VP5 plays a role in ensuring the release of mature IBD virions from the cell by lysis [7,8]. Segment B (2.8 kbp) encodes the RNA-dependent RNA polymerase VP1, which is involved in replication and transcription as well as contributing to viral virulence [11].

Based on pathogenicity, two serotypes of IBDV are known: serotype 1, which is pathogenic to chickens, and serotype 2, a nonpathogenic strain [12]. Serotype 1 consists of four groups: classical, antigenic variant, very virulent, and attenuated [13,14]. As an RNA virus, IBDV is prone to mutation and recombination, culminating in reassortant(s) and recombinant(s) [15,16]. The classification of IBDV was initially based on the different strains that existed—namely, classical, variant, and very virulent. However, Michel and Jackwood [17] proposed the classification of IBDV serotype 1 into seven genogroups (G1–G7) based on the VP2 hypervariable region sequences. Recently, Islam et al. [18] proposed a further classification based on the phylogenetic analysis of both segments A and B. Two genetic fragments were considered to build the dataset: a 366 bp portion of segment A that includes the VP2 hypervariable region (nt 785–1150), and a 508 bp fragment of segment B focused on VP1 N-terminal domain and the finger subdomain of the central polymerase (nt 328–835). This classification produced eight genogroups for segment A (A1–A8), with serotype 2 designated as A0, and five genogroups (B1–B5) for segment B, with serotype 2 designated as B1. Another classification by Wang et al. [19] identified nine genogroups for segment A (A1–A9) and five (B1–B5) for segment B.

In Nigeria, IBD is endemic, with reports of outbreaks in vaccinated poultry flocks [4,20,21]. In most IBD outbreaks in Nigeria, the very virulent (vv) IBDV strain has been implicated [20,21,22,23,24,25,26]. In 2016, the presence of reassortant IBDV was first confirmed in Nigeria [21]. It has been speculated that the reassortant viruses arose from the exchange of genetic materials between the very virulent field and attenuated (vaccine) strains of IBDV. The presence of reassortant viruses is one of the factors that can give rise to vaccination failure, and, therefore, their detection could be one of the reasons for IBDV vaccination failure in Nigeria. The presence of the vvIBDV and the reassortant IBDV have been associated with the failure of IBDV vaccination even under strict management practices [27].

Here, we sequenced the near-complete genomes of four IBDV collected between 2017–2019 with the aim of gaining insight into the evolutionary dynamics of the virus. This information is critical to developing an effective control strategy to reduce the threat of the disease.

## 2. Materials and Methods

### 2.1. Sources of Samples

Suspected outbreaks of IBD from four different geographical locations in Nigeria were investigated from 2017 to 2019. Epidemiological information on the outbreaks were documented. The number of birds per flock ranged from 350 to 30,000. In each of the outbreak locations, 3–5 carcasses were collected from the farm for investigation. At postmortem, 3–5 bursae of Fabricius (bF) were collected from birds within the same outbreak location for processing as a pool.

### 2.2. IBD Virus Antigen Detection

The bF was processed into 20% (*w*/*v*) homogenate as previously described [25]. The supernatant was analyzed using the agar gel precipitin test (AGPT) alongside standard IBDV antigen and antiserum (Charles Rivers Laboratories, Wilmington, NC, USA). The agar plates were incubated in a humidified incubator at 37 °C for 48 h. Aliquots of the supernatant were stored at −80 °C until shipped to Istituto Zooprofilattico Sperimentale delle Venezie, Padua, Italy for molecular analysis.

### 2.3. Sequencing

According to the manufacturer’s instructions, total RNA was extracted from the bursal homogenate samples using the QIAsymphony RNA kit (Qiagen, Hilden, Germany). RNA from one of the four samples was reverse-transcribed into double-stranded cDNA (cDNAds) using Maxima H Minus Double-stranded cDNA synthesis kit (ThermoFisher, Waltham, MA, USA) to be sequenced using a metagenomic approach. RNA from the other three samples was reverse-transcribed and amplified using SuperScript™ III One-Step RT-PCR System with Platinum™ Taq High Fidelity DNA Polymerase (ThermoFisher, Waltham, MA, USA) and specific primers to amplify segment A (IBDV-VV_A-83-F 5′-AACTCCTCCTTCTACAATGC-3′; IBDV-VV_A-3123-R 5′-CTGTGTTGGAGCATTGGG-3′) and segment B (IBDV-VV_B-91-F 5′-TCTTCTTGATGATTCTRCCAC-3′; IBDV-B-2782-R 5′-TCTTCTTGAGTGGTTCCC-3′). Double-stranded cDNA and amplicons were purified using Agencourt AMPure XP (Beckman Coulter™) and quantified with Qubit™ DNA HS Assay (Thermo Fisher Scientific, Waltham, MA, USA). Sequencing libraries were generated using the Illumina Nextera XT DNA Sample Preparation Kit (Illumina, San Diego, CA, USA) following the manufacturer’s instructions and sequenced using the Illumina MiSeq platform (2 × 300 bp PE mode).

### 2.4. Bioinformatic Analysis

Illumina read quality was assessed using FastQC v0.11.2. Raw data were filtered by removing (a) reads with more than 10% of undetermined (“N”) bases; (b) reads with more than 100 bases with Q score below 7; and (c) duplicated paired end reads. The remaining reads were clipped from Illumina adaptors with Scythe v0.991 (https://github.com/vsbuffalo/scythe (accessed on 6 May 2019)) and, only for samples processed with the target amplification approach, from PCR primers with Trimmomatic v0.32 [28], before being trimmed with Sickle v1.33 (https://github.com/najoshi/sickle (accessed on 6 May 2019)). Reads shorter than 80 bases or unpaired after previous filters are discarded.

Reads associated with the sample sequenced using the metagenomic approach were further processed by taxonomic assignment using BLAST v2.10.0+ [28] alignment against the integrated NT database (version 23 February 2020) and Diamond v0.9.17 [29] alignment against the integrated NR database (version 23 February 2020). Alignment hits with e-values greater than 1 × 10^−3^ were discarded. The taxonomical level of each read was determined by the lowest common ancestor-based algorithm implemented in MEGAN v6.18.50 [30]. Reads not classified taxonomically as belonging to IBDV were discarded.

High quality reads that passed previous filters were aligned against the IBDV reference genome (for the samples sequences with target amplification approach: AF051837.1 for segment A, AB368969.1 for segment B; for the sample sequenced with the metagenomic approach: AF092943.1 for segment A, KF569804.1 for segment B) using BWA v0.7.12 [31] and standard parameters. Alignments were processed using SAMtools v0.1.19 [32] to convert them into BAM format and sort them by position.

SNPs were called using LoFreq v2.1.2 [33]. According to LoFreq usage recommendations, the alignment was first processed with Picard-tools v2.1.0 (http://picard.sourceforge.net (accessed on 23 February 2020)) and GATK v3.5 [34] in order to correct potential errors, realign reads around indels, and recalibrate base quality. LoFreq was then run on fixed alignment with an option “-call-indels” to produce a vcf file containing both SNPs and indels. From the final set of variants, indels with a frequency lower than 50% and SNPs with a frequency lower than 25% were discarded. To produce the consensus sequence, we changed the reference genome in agreement with the following rules: (a) for a position j, and if coverage was not high enough (<10×) to make reliable call variants, we added an “N” base; (b) for a position j, and if coverage was high enough to make reliable call variants but no SNP was called, we added reference genome base at position j; and (c) for a position j, and if coverage was enough to make reliable call variants and at least one SNP was called, we added the nucleotide using the IUPAC nucleotide code (http://www.bioinformatics.org/sms/iupac.html (accessed on 23 February 2020)) in accordance with bases present.

### 2.5. Phylogenetic Analysis

The phylogenetic analysis was carried out following the criteria suggested by Islam et al. [18]. More specifically, for the VP2 hypervariable region (366 bp) of segment A, the dataset included (i) 151 sequences representative of the eight genogroups of serotype 1; (ii) sequences obtained from BLAST results; and (iii) one genogroup of serotype 2. Serotype 1 sequences included: A1a (classical attenuated) and A1b (classical virulent), A2 (US antigenic variant), A3 (very virulent), A4 (early European and recent South American distinct IBDV, dIBDV), A5 (atypical or recombinant Mexican), A6 (atypical Italian), A7 (early Australian), and A8 (Australian variant). The dataset for segment B of the VP1 N-terminal domain and the finger subdomain of the central polymerase fragment (508 bp) includes 134 sequences representative of the 5 distinct genogroups, namely, B1 (classic-like), B2 (very virulent-like), B3 (early Australian-like), B4 (Polish & Tanzanian), B5 (Nigerian), and sequences obtained from the BLAST search. Alignment for both datasets was performed using FFT-NS-i (Standard) implemented in MAFFT V.7.0 (https://mafft.cbrc.jp/alignment/server/ (accessed on 15 June 2022) [35]. The phylogenetic trees were obtained using IQ-TREE 1.6.10 [36] (version 20 March 2019), with 1000 ultra-fast bootstrap replicates and the best fit model automatically identified by the software; in detail, for segment A phylogenetic tree, the best-fit model was chosen according to BIC was SYM+I+G4, whereas the GTR+F+G4 model was automatically selected for segment B. The FigTree v.1.4.3 (https://github.com/rambaut/figtree/releases (accessed on 6 May 2019)) program was used to view phylogenetic trees graphically.

## 3. Results

### 3.1. Flock History

**Figure 1:**
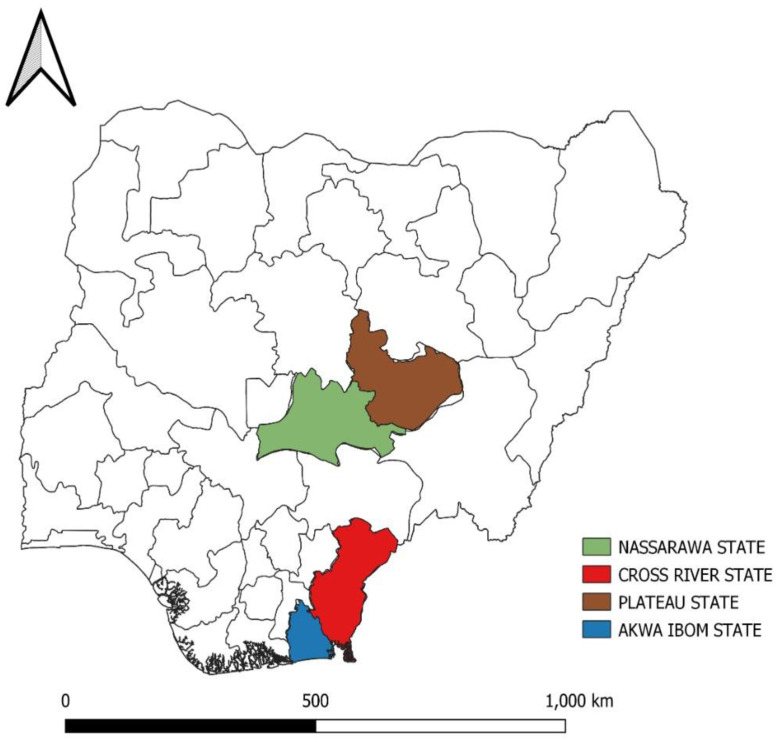
Map of Nigeria showing the locations where the samples were obtained for the study.

The locations where the IBD outbreak investigations were carried out were Nasarawa, Plateau (Northcentral), Cross River, and Akwa Ibom (South-south) States (Figure 1) in Nigeria. Epidemiological information on the outbreaks is shown in Table 1.

### 3.2. Detection of IBD Viruses

In all the samples (*n* = 15) tested by AGPT, precipitin bands were observed between 24 to 48 h post-incubation. Only 4 samples identified as being from different outbreaks were considered for sequencing. The sequences have been submitted to the GenBank database under accession numbers OP311682–OP311689.

### 3.3. Phylogenetic Analysis

**Figure 2:**
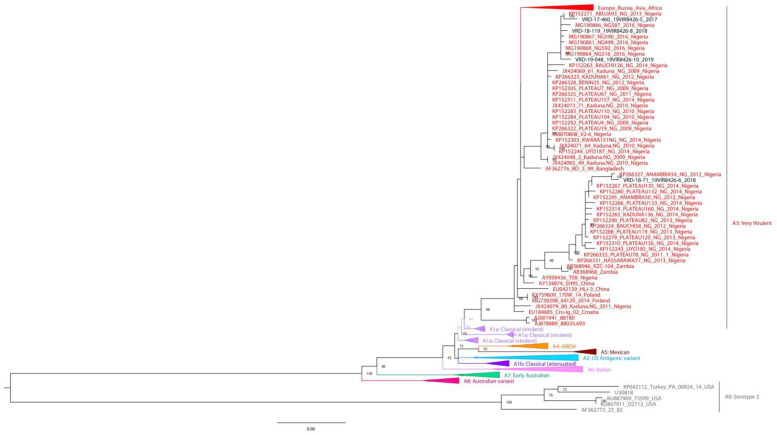
Phylogenetic tree of nucleotide sequences of partial segment A of IBDV based on VP2 hypervariable region. Maximum likelihood method with the best-fit model (SYM+I+G4) automatically identified by IQ-TREE v.1.6.12. All genogroups except A3 and A0 have been collapsed. The samples generated in the study are identified in black. The ultra-fast bootstrap values higher than 70% are indicated near the nodes.

The phylogenetic tree of the VP2 fragment (366 bp) from segment A shows that all four samples clustered into A3 genogroup (Figure 2), which contains very virulent pathotypes, and are closely related to viruses collected between 2009 and 2016 in Nigeria. In particular, sample VRD-18-71_19VIR8426-6 clustered with strains collected in Nigeria from 2011 to 2014, showing the highest nucleotide sequence identity with the Nigerian virus ANAMBRA54/NG/2012 (99.2%); samples VRD-17-460_19VIR8426-5, VRD-18-119_19VIR8426-8 and VRD-19-048_19VIR8426-10 cluster together with Nigerian IBDV samples collected from 2009 to 2016; and, in detail, VRD-17-460_19VIR8426-5 shows the highest nucleotide sequence identity (98.9%) with ABUJA93/NG/2013, while VRD-18-119_19VIR8426-8, and VRD-19-048_19VIR8426-10 are closely related to NG592/2016 (99.4% and 99.2%, respectively).

**Figure 3:**
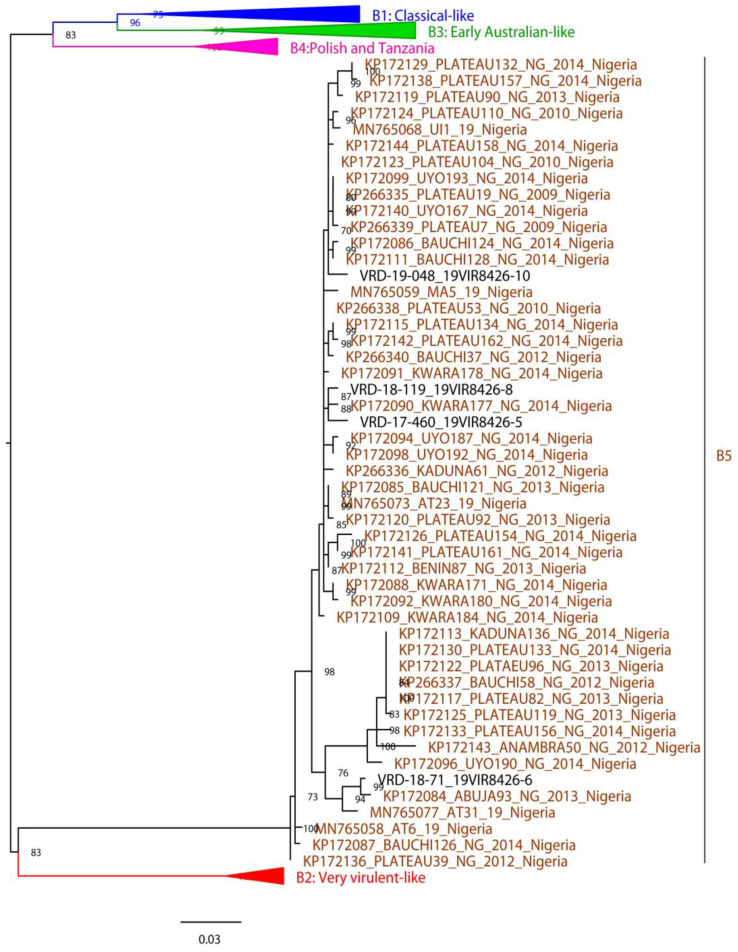
Phylogenetic tree of nucleotide sequences of partial segment B of IBDV based on VP1 N-terminal domain and finger subdomain of central polymerase. Maximum likelihood method with the best-fit model (GTR+F+R4) automatically identified by IQ-TREE v.1.6.12. All genogroups except B5 (brown color) have been collapsed. The ultra-fast bootstrap values higher than 70% are indicated near the nodes.

The phylogenetic tree based on partial segment B sequence (508 nt) (Figure 3) shows that all four samples are grouped in the B5 genogroup, with Nigerian samples circulating in 2009–2019. Sample VRD-17-460_19VIR8426-5 and VRD-19-048_19VIR8426-10 show the highest identity (99.1%) with samples isolated in Nigeria in 2010–2014, VRD-18-119_19VIR8426-8 with PLATEAU53/NG/2010 (99.4%), and sample VRD-18-71_19VIR8426-6 with ABUJA93/NG/2013 (99.4%).

### 3.4. Nucleotide Sequence Analysis of Segments A and B

The consensus sequences obtained were compared to the GenBank database using the BLAST algorithm (https://blast.ncbi.nlm.nih.gov/Blast.cgi (accessed on 15 June 2022)), and the results are summarized in Table 2.

For the four Nigerian IBDV isolates examined in this study, nucleotide analysis of the complete segment A (VP2-4-3 polyprotein) and segment B (VP1) to each other range was from 94.9–99.1% and 97.1–98.8%, respectively.

### 3.5. Amino Acid Sequence Analysis of Segments A and B

Amino acid sequences of both segments of the four Nigerian isolates were compared with full and partial sequences of some previously published Nigerian IBDVs, with representative IBDV sequences from GenBank comprising very virulent, variant, attenuated and reassortant strains. In all, thirty-one amino acid substitutions were observed in the VP5 of the three Nigerian isolates, with six of these substitutions being unique only to Nigerian IBDVs (Table S1). The polyprotein (VP2-VP4-VP3) had thirty-five substitutions (Table S2), four of which were unique to the Nigerian IBDVs. The VP1 had a total of twenty substitutions, four of which were unique to Nigerian IBDVs.

The VP2 amino acid sequences of the four Nigerian IBDVs under investigation had the markers typical of vvIBDV (222A, 242I, 256I, 294I and 299S) [37,38,39]. The serine-rich heptapeptide region (SWSASGS) spanning from amino acid position 326 to 332 adjacent to the second hydrophilic region of the VP2 gene were also conserved, as established in other vvIBDVs. At amino acid position 300, the substitution E→A was observed in three of the four Nigerian IBDVs examined, while the fourth had the substitution E→Q. Amino acid substitution at position 300 has been implicated for vaccination failure [21,23].

Analysis of the VP4 amino acid sequences of the four Nigerian IBDV and others from GenBank revealed H471Q and Q486L mutations in three of the four Nigeria IBDV previously reported [23]. Interestingly, the Nigerian IBDV, VRD-18-71_19VIR8426-6, had unique amino acid mutations F599Y, D614E, and K642Q (Table S2) that were not observed in the other three IBD viruses under investigation nor previously reported in IBDV from Nigeria. Another substitution, N745S, was observed in three of the four Nigerian IBD viruses and two previously reported Nigerian IBDVs used for comparison and in a reassortant IBDV from China (HLJ-0504).

Sequence analysis of VP3 revealed amino acid substitutions E761G (Table S2) in three of the four Nigerian IBD viruses. These substitutions were also present in some previously reported Nigerian IBD viruses [23]. Position 767 of one of the four Nigerian IBD viruses had this mutation, S767G, which was also present in the previously reported Nigerian IBDV, T09 [22]. At amino acid positions 777 and 778, the following substitutions, N→S and V→A, were only seen in VRD-19-048_19VIR8426-10. The mutation at position V990A (Table S2) was observed in all four Nigerian IBDVs.

Sequence analysis of the VP1 of the four Nigerian IBDV used in this study revealed the following mutations: V4I in one of four (as in other IBDVs, notably attenuated and reassortant strains used for comparison), T23S in three of the four, and E119D and V141I in all of the four. The triplet amino acid at positions 145, 146, and 147 (Table S3) of the four Nigerian IBDVs showed similar QEG mutations, as previously reported [21]. Positions 150 and 158 had D→E and N→S mutations, with some IBDV from China and Europe, respectively. At position 163, the substitution A→V was observed in all the Nigerian IBDV examined and a reassortant IBDV strain GX-NNZ-11, while the following mutations at positions D219E, E242D, M390L, A391T, D393E and P562S were observed in all four (Table S3). The substitution, R695K, was observed in three of the four Nigerian IBD viruses. At position 697, V→T occurred in three out of four while Nasarawa 8426-8/2018 Nigeria had V→I and Cross River/19VIR8426-6/2018 only had the K761R mutation (Table S3).

## 4. Discussion

This study provided insight into the sequences from four near-complete genomes of IBDV from different outbreaks and locations in Nigeria. Thanks to deep sequencing results, it was possible to inspect SNP present in segment A and B and to assess that they do not support the presence of multiple variants for each segment belonging to the IBDV. Unique amino acid mutations with as yet unknown biological functions have been identified. Most of the previous molecular studies on IBDVs in Nigeria are based on partial sequence analyses of either the hypervariable (hv) VP2 or the VP2 and VP1 genes [20,21,22,23,24,26]. As of February 2023, only two complete genome sequences of segment A [22,23] and segment B [22] of Nigerian IBDV exist in the GenBank. Sequence analysis of both VP2 and VP1 has been advocated to determine the virulence of IBDV and to identify genetic reassortments [15,40,41]. The complete genome sequence analysis of IBDV has helped to advance our understanding of the dynamics, epidemiology, and evolution of IBDV to aid in its prevention and control [42,43,44]. This study has, therefore, further elucidated the evolution of IBDV in Nigeria.

Phylogenetic analysis using the nucleotide sequences of the four Nigerian IBDVs studied showed that they are reassortant viruses. Previous studies in the country have identified reassortant IBDV strain, with segment A derived from vvIBDV and segment B derived from non-vvIBDV [21,26]. The previously identified reassorted viruses produced a novel lineage unique to Nigeria [21] and, recently, Islam et al. [18] re-assigned the Nigerian reassortant IBD viruses into a new group B5. Based on the hypervariable region of the VP2, all four IBDV belong to the very virulent strains. Previous molecular studies on IBDV in Nigeria reported the presence of the vvIBDV strains circulating in poultry [20,21,22,23,24,25,26]. Using the criteria for classifying IBD viruses proposed by Michel and Jackwood [17], Nigerian viruses clustered with the G3 group of IBDV. However, based on the recent IBDV classification by Islam et al. [18], Nigerian IBDVs exclusively formed a monophyletic cluster designated as A3B5 (where A3: IBDV isolate with vvIBDV-like segment A and B5: IBDV isolate with non-vvIBDV-like segment B).

The high percentage identity of the polyproteins and VP5 (94.9–99.5%) of the three studied viruses with each other and the previously reported Nigerian IBDVs might indicate a relative level of stability of the virus within these regions in Nigeria, albeit not at 100% compared to previously reported IBDVs from Nigeria, as IBDV is well known for its ability to mutate and evolve [25]. The results of the nucleotide sequence analysis of segment B of the four Nigerian IBDV showed a low percentage nucleotide identity with a previously reported Nigerian IBDV (T09), UK661, and even the classic D78. The former viruses are vvIBDV, with segments A and B derived from vvIBDV-like viruses, while the latter, a classic IBDV, has segments A and B derived from non-vvIBDV-like viruses.

In the amino acid sequences of segment A of the four Nigerian IBDV isolates used in this study, the hvVP2 region, extending from amino acid 206–350, which is responsible for tissue culture adaptation, antigenic variation, and antigenicity, exhibited the markers typical of vvIBDV [37,38,39]. The serine-rich heptapeptide region from aa326–332 was also conserved in all four, indicating that they belong to the vvIBDV [45]. Within this region, few previously reported amino acid substitutions Q219T [21,26], G254S, T269S, and E300A/Q [21,22,23,26] were observed in a single, some, or all of the four Nigerian viruses. The occurrence of three of these mutations (Q219T, G254S and E300A/Q) within the hydrophilic loops of the virus can affect their antigenicity, which, in turn, can lead to a drift [46]. The four Nigerian IBDVs also have these amino acid residues, I272, M290, Q324 and S330, which are intrinsic to vvIBDV [47,48]. Apart from the delineated amino acid substitutions within the hvVP2 region of the four Nigerian IBDVs, the amino acid VP2 sequences from aa1–452 were conserved as observed in the vvIBDVs.

The VP4 amino acid sequences of the four Nigerian viruses had amino acids that were conserved for vvIBDV [41], except at position 685, where three out of the four Nigerian IBDVs had N (asparagine) replaced with S (serine). Within the Nigerian IBDV VP4, other amino acid substitutions occurred at the following positions: H471Q, Q486L, F599Y, D614E, K642Q, N745S, in either a single, some, or all four of the viruses. These substitutions were also observed in some previously reported IBDV from Nigeria (Table S2). Apart from being the viral protease responsible for the cleavage of the polyprotein, VP4 plays a vital role in IBDV replication, growth, and maturation [49]. Therefore, any variation observed within this region may affect the virulence of the virus [48,50]. These mutations observed in the VP4 sequence of the Nigerian viruses need further investigation to determine their effect on viral pathogenicity.

The VP3, one of the major structural proteins of IBDV, acts as a scaffolding protein that binds the viral double-stranded RNA and, in collaboration with VP1, mediates recovery from infectious IBDV [51]. The VP3 may also play a role in receptor-mediated virus-cell attachment and virulence of IBDV [52]. The four Nigerian IBDVs examined had five amino acid substitutions (Table S2) compared to the vvIBDVs. Some of the mutations were observed in previously reported IBDV from Nigeria, except for the following substitutions: N777S and V778A, which were only seen in Akwa Ibom/19VIR8426-10/2019. The substitution A990 found in non-vvIBDVs was observed in the four Nigerian IBDVs. This mutation (A990) has been reported to decrease IBDV replication and efficacy during challenge [53].

Apart from the amino acid substitutions observed in the three Nigerian IBDV (Table S1), other amino acids within the VP5 were conserved, as seen in vvIBDVs. Two Nigerian isolates, Nasarawa/19VIR8426-8/2018 and Plateau/19VIR8426-5/2017 VP5 amino acid sequences, started at position 16, while that of Akwa Ibom/19VIR8426-10/2019 was at position 2. Amino acid sequences of vvIBDV VP5 are 149 long, while those of non-vvIBDV are 145 long [54]. Some of the substitutions observed in the three Nigerian viruses are novel compared to other previously reported Nigerian IBDVs. Some of the mutations do not correspond with the positions mapped on the VP5 to differentiate the vvIBDV strains from other strains. The IBDV VP5, a non-structural protein, although not essential for viral replication, plays an essential role in its pathogenesis, prevents apoptosis, and plays a role in the adaptive evolution of IBDV [10,55,56]. The role of the mutations observed in the three Nigerian IBDVs in terms of pathogenicity is still unknown.

The complete genome sequence of the VP1 of the four Nigerian IBDVs has advanced our understanding of the evolution of reassortant IBDVs in Nigeria. Currently, there is only one full-length genome sequence of the Nigerian IBDV VP1 in the GenBank [22]; others are partial sequences. Reassortment events have been associated with vaccine failure in IBDV [27]. In addition, the VP1 of IBDV plays a role in the pathogenicity of the virus, an event previously attributed only to VP2 [57]. However, reassortant IBDVs have been reported in Nigeria [21,26], and those studies used partial sequences of the VP1 to arrive at their conclusions. However, the only full-length sequence of the Nigerian IBDV VP1 sequence in the GenBank [22] is not a reassortant IBDV. Hence, this present study provided insight into a complete VP1 reassortant IBDV from Nigeria. The recent Nigerian IBDVs VP1 showed some of the previously reported mutations that are unique to Nigerian IBDVs, including others that were not reported previously because the partial sequences of the VP1 were used in the analysis. The vaccination regimen in Nigeria comprises administration of at least two different doses of IBDV vaccines at the early stages of production. In three of the four investigated cases, IBDV was administered twice, and outbreak was still recorded. The reason for the possible vaccine failure could not be fully established but may be associated with the circulation of reassortant IBDVs. Future studies may be geared towards evaluation of the current vaccination schedule against the reassortant viruses in a vaccination-challenge experiment.

As reported by Cui et al. [58], sixteen amino acids are conserved for vvIBDVs in the VP1. Analysis of the VP1 amino acid sequences of the four Nigerian viruses revealed twenty-one (Table S3) amino acid substitutions compared to vvIBDV UK661. Eight of the amino acid substitutions were unique to the four Nigerian IBDV isolates. The implication of these mutations on the pathogenicity of the virus merit further investigation.

To better understand the evolutionary dynamics of the VP1 of Nigerian viruses, the VP1 amino acid sequences of the four Nigerian IBDVs used in this study were aligned and compared to the partial VP1 sequences of previous Nigerian reassortant IBDVs (aas 26–299). A total of eleven amino acid substitutions were observed between the recent VP1 amino acids and the previously reported VP1 amino acids. The substitutions observed in the previously reported Nigerian IBDV VP1 amino acids were not seen in the recent ones used for this study, suggesting the possible evolution of the virus.

## 5. Conclusions

The near-complete genome sequences of four Nigerian Infectious bursal disease viruses were determined and molecular markers typical of very virulent IBDV strains were identified. Likewise, segment B of the four Nigerian viruses further confirmed the presence of reassortant strains of IBDV with a combination of this unique amino acid combination QEG at the triplet positions (145–147), which appears to be stable since its first report in Nigeria. These recent IBDVs identified five new amino acid mutations compared to previously reported IBDVs from Nigeria, confirming the continuous evolution of IBDV in Nigeria. This study also highlights the need for constant epidemiological investigation of IBDV to keep track of any new or sudden changes over time as these may impact vaccine efficiency.

## Figures and Tables

**Table 1:** Epidemiological information on the IBD outbreaks from the investigated locations in Nigeria.

Virus Id	Year	Flock Size	Outbreak Location	Specie	Vaccination Status
VRD/17/460_19VIR8426-5	2017	350	Plateau	Chicken	Not vaccinated
VRD/18/71_19VIR8426-6	2018	30,000	Cross-River	Chicken	IBDV twice
VRD/18/119_19VIR8426-8	2018	5000	Nasarawa	Chicken	IBDV twice
VRD/19/048_19VIR8426-10	2019	3000	Akwa-Ibom	Chicken	IBDV twice

**Table 2:** Closest BLAST matches against NCBI database of IBDV segments A and B.

Virus ID	Segment	Accession Number	% Similarity	Description	Accession Number	Country
VRD/17/460_19VIR8426-5	A	OP311683	98.08	IBDV 80/Kaduna.NG/2011	JX424079.1	Nigeria
	B	OP311687	90.09	IBDV strain 150133/3.2	MF969112.1	Algeria
VRD/18/71_19VIR8426-6	A	OP311684	96.61	IBDV strain T09	AY099456.1	Nigeria
	B	OP311688	90.43	IBDV HuB-1	GQ449693.1	China
VRD/18/119_19VIR8426-8	A	OP311685	98.08	IBDV 80/Kaduna.NG/2011	JX424079.1	Nigeria
	B	OP311689	90.20	IBDV strain 150133/3.2	MF969112.1	Algeria
VRD/19/048_19VIR8426-10	A	OP311682	98.16	IBDV 80/Kaduna.NG/2011	JX424079.1	Nigeria
	B	OP311686	92.91	IBDV strain 150133/3.2	MF969112.1	Algeria

## Data Availability

All data that supports the findings of this study are available in the main manuscript and the Supplementary Materials (Table S1: Amino acid substitutions at the VP5; Table S2: Amino acid substitutions at the VP2, VP4 and VP3; Table S3: Amino acid substitutions at the VP1). The sequence data can be found under the accession numbers OP311682–OP311689.

## Supplementary Materials

The following supporting information can be downloaded at: https://www.mdpi.com/article/10.3390/vaccines11040867/s1, Table S1: Amino acid substitutions at the VP5; Table S2: Amino acid substitutions at the VP2, VP4 and VP3; Table S3: Amino acid substitutions at the VP1.
