# Supplementary material for: Genomic Analysis of Infectious Bursal Disease Virus in Nigeria: Identification of Unique Mutations of Yet Unknown Biological Functions in Both Segments A and B
Source: Vaccines (Basel). 2023 Apr 19;11(4):867. doi: 10.3390/vaccines11040867 (PMC10144922; doi:10.3390/vaccines11040867)
Supplement: Supplementary file 1 [file vaccines-11-00867-s001.zip › vaccines-2312730-supplementary.pdf]

Table S1. Amino acid substitutions at the VP5

STRAIN	PHENOTYPE	VP5												
		21	31	44	45	53	80	104	108	109	116	119	122	138
AYA57922.1 UK661	Very virulent	R	T	S	G	S	W	C	T	G	A	S	H	H
Plateau 8426-5/2017 Nigeria	ND	G	S	T	R	T	G	G	A	S	T	.	.	N
Cross River 8426-6/2018 Nigeria	ND													
Nasarawa 8426-8/2018 Nigeria	ND	.	S	T	R	T	G	G	A	.	T	P	D	N
Akwa Ibom 8426-10/2019 Nigeria	ND	.	S	T	R	T	G	G	A	.	T	.	.	N
AAM45384.1 T09 Nigeria	Very virulent	.	.	.	.	.	.	.	.	.	.	.	.	.
AFU10478.1 NIGERIA	Very virulent	.	.	.	.	T	.	.	.	.	.	.	.	N
AFU10443.1 NIGERIA	Very virulent	.	.	.	.	T		.	.	.	T	.	.	.
AMQ81722.1 MB11 India	Attenuated	.	.	.	.	.	.	.	.	.	.	.	.	.
AFX62899.1 HN04 China	Reassortant	.	.	.	.	.	.	.	.	.	.	.	.	.
AFP86286.1 GX-NNZ-11 China	Reassortant	.	.	.	.	.	.	G	.	.	.	.	.	.
ACZ62645.1 HLJ-0504 China	Reassortant	.	.	.	.	.	.		.	.	.	.	.	.
ANY95160.1 THI14/ABT/MVC/India		.	.	.	.	.	.	G	.	.	.	.	N	.
ANY95157.1 VCN14/ABT/MVC/India		.	.	.	.	.	.	.	.	.	.	.	.	.
AMQ24274.1 BGE14/ABT2/MVC/2015 INDIA	Reassortant	.	.	.	.	.	.	.	.	.	.	.	.	.
AAO15768.1 D78	Attenuated	.	.	.	.	.	.	.	.	.	.	.	.	.
AAD32617.1 VARIANT E	Variant	.	.	.	.	.	.	.	.	.	.	.	.	.
AYA57922.1 D6948	Very virulent	.	.	.	.	.	.	.	.	.	.	.	.	.
AVH80604.1 IBDV/ITALY/1829/2011		.	.	.	.	.	.	.	.	.	.	.	.	.
AGW24627.1 SOUTH AFRICA												.	.	.
AUS90754.1 16002 EGYPT		.	.	.	.	.	.	.	.	.	.	.	.	N

Table S2. Amino acid substitutions at the VP2, VP4 and VP3

Strain	Phenotype	VP2						VP4					VP3			
		219	254	269	300	471	486	599	614	642	685	745	761	767	777	778
AYA57922.1 UK661	Very virulent	Q	G	T	E	H	Q	F	D	K	N	N	E	S	N	V
Plateau 8426-5/2017 Nigeria	ND	.	S	.	A	Q	L	.	.	.	S	S	G.	.	.	.
Cross River 8426-6/2018 Nigeria	ND	T	S	S	Q	.	.	Y	E	Q	.	.	.	G	.	.
Nasarawa 8426-8/2018 Nigeria	ND	.	S	.	A	Q	L	.	.	.	S	S	G	.	.	.
Akwa Ibom 8426-10/2019 Nigeria	ND	.	S	.	A	Q	L	.	.	.	S	S	G	.	S	A
AAM45384.1 T09 Nigeria	Very virulent	.	.	.	.	.	.	.	.	.	.	.	.	G	.	.
AFU10478.1 NIGERIA	Very virulent	.	.	.	.	Q	L	.	.	.	S	S	G	.	.	.
AFU10443.1 NIGERIA	Very virulent	.	S	.	A	Q	L	.	.	.	S	S	G	.	.	.
AMQ81722.1 MB11 India	Attenuated	.	.	.	.	.	.	.	.	.	K	.	.	.	.	.
AFX62899.1 HN04 China	Reassortant	.	.	.	.	.	.	.	.	.	K	.	.	.	.	.
AFP86286.1 GX-NNZ-11 China	Reassortant	.	.	S	.	.	.	.	.	.	K	.	.	.	.	.
ACZ62645.1 HLJ-0504 China	Reassortant	.	.	.	.	.	.	.	.	.	.	S	.	.	.	.
ANY95160.1 THI14/ABT/MVC/India		.	.	.	.	.	.	.	.	R	.	.	.	.	.	.
ANY95157.1 VCN14/ABT/MVC/India		.	.	.	A	.	.	.	.	.	K	.	.	.	.	.
AMQ24274.1 BGE14/ABT2/MVC/2015 INDIA	Reassortant	.	.	.	A	.	.	.	.	.	.	.	.	.	.	.
AAO15768.1 D78	Attenuated	.	.	.	.	.	.	.	.	.	K	.	.	.	.	.
AAD32617.1 VARIANT E	Variant	.	S	.	.	.	.	.	.	.	K	.	.	.	.	.
AYA57922.1 D6948	Very virulent	.		.	.	.	.	.	.	.		.	.	.	.	.
AVH80604.1 IBDV/ITALY/1829/2011		.	S	S	.	.	.	.	.	.	K	.	.	.	.	.
AGW24627.1 SOUTH AFRICA		.	S	.	.	.	.	.	.	.		.	.	.	.	.
AUS90754.1 16002 EGYPT		.	S	.	.	.	.	.	.	.		.	.	.	.	.

Table S3. Amino acid substitutions at the VP1

STRAIN	PHENOTYPE	VP1															
		990	4	23	119	141	145	146	147	150	158	163	219	242	269	390	391
AYA57922.1 UK661	Very virulent	V	V	T	E	V	T	D	N	D	N	A	D	E	E	M	A
Plateau 8426-5/2017 Nigeria	ND	A	.	S	D	I	Q	E	G	E	S	V	E	D	D	L	T
Cross River 8426-6/2018 Nigeria	ND	A	.	S	D	I	Q	E	G	.	.	V	E	D	D	L	T
Nasarawa 8426-8/2018 Nigeria	ND	A	I	.	D	I	Q	E	G	E	.	V	E	D	D	L	T
Akwa Ibom 8426-10/2019 Nigeria	ND	A	.	S	D	I	Q	E	G	E	.	V	E	D	D	L	T
AAM45384.1 T09 Nigeria	Very virulent	.	.	.	.	.	.	.	.	.	.	.	.	.	.	.	.
AFU10478.1 NIGERIA	Very virulent	A	-	-	-	-	-	-	-	-	-	-	-	-	-	-	.
AFU10443.1 NIGERIA	Very virulent	A	-	-	-	-	-	-	-	-	-	-	-	-	-	-	.
AMQ81722.1 MB11 India	Attenuated	A	I	.	.	.	N	E	G	.	.	.	.	D	.	L	.
AFX62899.1 HN04 China	Reassortant	A	I	.	.	.	N	.	.	.	.	.	.	.	.		.
AFP86286.1 GX-NNZ-11 China	Reassortant	A	I	.	.	I	S	E	D	.	.	V	.	D	.	L	.
ACZ62645.1 HLJ-0504 China	Reassortant	A	.	.	.	L	.	E	G	.	.	.	.	D	.	L	.
ANY95160.1 THI14/ABT/MVC/India	Reassortant	A	I	.	.	.	N	E	G	.	.	.	.	D	.	.	.
ANY95157.1 VCN14/ABT/MVC/India	Reassortant	A	I	.	.	.	N	E	G	.	.	.	.	D	.	L	.
AMQ24274.1 BGE14/ABT2/MVC/2015 INDIA	Reassortant	A	I	.	.	.	N	E	G	.	.	.	.	D	.	L	.
AAO15768.1 D78	Attenuated	A	I	.	.	.	N	E	G	.	.	.	.	D	.	L	.
AAD32617.1 VARIANT E	Variant		.	.	.	.	N	E	G	.	.	.	.		.	L	.
AYA57922.1 D6948	Very virulent	.	.	.	.	.	.	.	.	.	.	.	.		.		.
AVH80604.1 IBDV/ITALY/1829/2011		A	I	.	.	.	.	E	G	.	.	.	.	D	.	L	.
AUS90754.1 16002 EGYPT		A	I	.	.	I	.	.	.	.	.	.	.	.	.	.	.

STRAIN	PHENOTYPE	VP1				
		393	562	695	697	761
AYA57922.1 UK661	Very virulent	D	P	R	V	K

Plateau 8426-5/2017 Nigeria	ND	E	S	K	T				
Cross River 8426-6/2018 Nigeria	ND	E	S		T	R			
Nasarawa 8426-8/2018 Nigeria	ND	E	S	K	I				
Akwa Ibom 8426-10/2019 Nigeria	ND	E	S	K	T				
AAM45384.1 T09 Nigeria	Very virulent	E	.	.	.	.			
AFU10478.1 NIGERIA	Very virulent	-	-	-	-	-	-	-	-
AFU10443.1 NIGERIA	Very virulent	-	-	-	-	-	-	-	-
AMQ81722.1 MB11 India	Attenuated	E	S	K	.	.			
AFX62899.1 HN04 China	Reassortant	V			.	.			
AFP86286.1 GX-NNZ-11 China	Reassortant	E	S	K	.	.			
ACZ62645.1 HLJ-0504 China	Reassortant	E	S	K	.	.			
ANY95160.1 THI14/ABT/MVC/India			S	K	.	.			
ANY95157.1 VCN14/ABT/MVC/India		E	S	K	.	.			
AMQ24274.1 BGE14/ABT2/MVC/2015 INDIA	Reassortant	E	S	K	.	.			
AAO15768.1 D78	Attenuated	E	S	K	.	.			
AAD32617.1 VARIANT E	Variant	E	S	K	.	.			
AYA57922.1 D6948	Very virulent	.	.	.	.	.			
AVH80604.1 IBDV/ITALY/1829/2011		E	S	.	.	.			
AGW24627.1 SOUTH AFRICA	-	-	-	-	-	-	-	-	-
